# Systemic Oxidative Stress in Subacute Stroke Patients Undergoing Rehabilitation Treatment

**DOI:** 10.3390/antiox13030354

**Published:** 2024-03-15

**Authors:** Carola Cocco, Mariacristina Siotto, Alessandro Guerrini, Marco Germanotta, Caterina Galluccio, Valeria Cipollini, Laura Cortellini, Arianna Pavan, Stefania Lattanzi, Sabina Insalaco, Elisabetta Ruco, Rita Mosca, Biagio Campana, Irene Aprile

**Affiliations:** 1 IRCCS Fondazione Don Carlo Gnocchi ONLUS, 50143 Florence, Italy; ccocco@dongnocchi.it (C.C.); alessandro.guerrini@unicampus.it (A.G.); mgermanotta@dongnocchi.it (M.G.); cgalluccio@dongnocchi.it (C.G.); vcipollini@dongnocchi.it (V.C.); lcortellini@dongnocchi.it (L.C.); apavan@dongnocchi.it (A.P.); slattanzi@dongnocchi.it (S.L.); sinsalaco@dongnocchi.it (S.I.); eruco@dongnocchi.it (E.R.); rmosca@dongnocchi.it (R.M.); bcampana@dongnocchi.it (B.C.); iaprile@dongnocchi.it (I.A.); 2 Department of Science and Technology for Humans and the Environment, Università Campus Bio-Medico di Roma, 00128 Rome, Italy

**Keywords:** oxidative stress, post stroke, rehabilitation, hydroperoxides, antioxidant defences, thiols

## Abstract

The imbalance in oxidative stress in acute stroke has been extensively studied; on the contrary, its investigation in the subacute phase is limited. The aim of this study was to analyse the variation in the systemic oxidative status in subacute post-stroke patients before (T0) and after a six-week rehabilitation treatment (T1) and to investigate the relationship between systemic oxidative status and rehabilitation outcomes. We enrolled 109 subjects in two different centres, and we analysed their serum hydroperoxide levels (d-ROMs), biological antioxidant power (BAP), thiol antioxidant components (-SHp), and relative antioxidant capacity (OSI and SH-OSI indices). Activity of Daily Living (ADL), hand grip strength, and walking endurance were evaluated using the modified Barthel Index, the Hand Grip test, and the 6-min walk test, respectively. At T0, most of the patients showed very high levels of d-ROMs and suboptimal levels of the BAP, OSI, and SH-OSI indices. Comparing the T1 and T0 data, we observed an improvement in the rehabilitation outcomes and a significant decrease in d-ROMs (549 ± 126 vs. 523 ± 148, *p* = 0.023), as well as an improvement in the OSI and SH-OSI indices (4.3 ± 1.3 vs. 4.7 ± 1.5, *p* = 0.001; 11.0 ± 0.4 vs. 1.2 ± 0.4, *p* < 0.001). In addition, significant correlations were seen between the oxidative stress parameters and the rehabilitation outcomes. These results suggest monitoring the systemic oxidative stress status in post-stroke patients in order to plan a tailored intervention, considering its relationship with functional recovery.

## 1. Introduction

Stroke is the main cause of adult disability [1,2] and the second main cause of death worldwide, which means it is consequently responsible for a significant cost for patients, families, and healthcare systems. Approximately 75% of all strokes occur in persons aged ≥65 years. As the population over 65 increases, it is anticipated that the incidence of strokes in older adults will rise, presenting significant challenges for medical professionals in the years to come [3].

Depending on the area of the brain affected and the extent of damage, stroke patients may present a heterogeneous clinical status. For this reason, a variable and often partial recovery of motor and cognitive function after rehabilitation treatment is also a possibility [4].

After an ischemic event, the resulting reduction in blood perfusion in the affected brain areas can result in the activation of numerous pathological cellular pathways, including inflammatory immune responses and increased oxidative stress through the formation of free radicals (primarily reactive oxygen and nitrogen species) [5,6].

Oxidative stress is defined as an imbalance between the production of free radicals and its scavenging by antioxidant defence molecules (both exogenous and endogenous), with a concomitant alteration in the redox circuits; it is also responsible for oxidative damage to macromolecules such as DNA, proteins, and lipids, resulting in the production of oxidative metabolites that persist in the systemic circulation [7,8,9]. Therefore, it is crucial to maintain a proper balance through the action of antioxidants with a high redox potential [10].

Oxidative stress is closely connected to the pathogenesis of cerebral stroke [11]; brain tissue is particularly vulnerable to oxidative damage, specifically lipid peroxidation, due to the high concentration of peroxidizable lipids and low levels of protective antioxidants at the level of the brain cells [11,12]. High levels of free radicals, such as peroxides, if not sufficiently counteracted by antioxidants, can generate a condition of general cellular damage that can trigger pro-apoptotic pathways, reducing the chance of neuron survival [13,14] and even being responsible for other stroke events [11].

After a stroke insult, increased levels of reactive oxygen species (ROS) in the brain have been linked to a reduction in brain plasticity, long-term synaptic potentiation, and synaptic signalling; these factors may be involved in the recovery of neurological deficits and neurorehabilitation [15,16].

Assessments of the oxidative stress acting directly on brain tissue in stroke animal models demonstrated an increase in oxidative markers and a reduction in the total antioxidant capacity during ischaemia and reperfusion in the acute phase of stroke [17,18,19]. Furthermore, they demonstrated a correlation between high levels of oxidative stress markers and worse neurological deficits in rats [19]. However, studies in animal models related to the subacute phase of stroke are limited.

The oxidative stress levels in the systemic circulation were found to be extremely high in post-stroke patients in the acute phase (1–7 days after a stroke event) [20,21,22,23]. Very few studies have investigated the oxidative stress in the subacute phase (within 6 months after a stroke event) [24,25,26] and the chronic phase of stroke (>6 months after a stroke event) [27,28], showing a similar imbalance.

Therefore, it would be important to find targeted therapies that can prevent oxidative damage to neuronal cells and preserve the correct redox balance in the brain tissue and the whole organism [5,29].

Regarding antioxidant status, a longitudinal study of the antioxidant levels in the first week after acute ischemic stroke showed that the mean plasma levels of nonenzymatic antioxidants (vitamin C, vitamin A, and vitamin E) and antioxidant enzyme activities (superoxide dismutase and glutathione peroxidase) were lower immediately after stroke compared to in healthy controls and then increased in the following days [30].

Among the main targets susceptible to ROS attack, there are also intracellular thiols [31]. As nucleophiles, the thiols present in peptides and proteins can be subjected to direct oxidation by ROS, causing structural and often functional protein changes. Furthermore, thiols contain a sulphydryl group (-SH), which participates in the thiol/disulphide redox couple, an efficient antioxidant system. This functional group is especially present in glutathione, thioredoxins, and various proteins, most notably albumin, which is rich in cysteine residues [32]. The recent literature has shown that following a stroke event, the levels of thiols are reduced [33,34].

A previous study from our group [24] found that subacute patients had an altered oxidative status on admission to a rehabilitation program; moreover, men showed a correlation between ROS and functional outcome.

Systemic oxidative status variation in subacute stroke patients during rehabilitation treatment has not been extensively studied. To our knowledge, few studies have examined this topic [35,36,37,38].

The aim of this study was then to investigate the following, in a cohort of post-stroke patients, during a 6-week rehabilitation treatment: (i) the systemic oxidative stress, including antioxidant–thiol compounds, on admission and after the rehabilitation treatment; (ii) the relationship between the systemic oxidative status and rehabilitation outcomes.

## 2. Materials and Methods

### 2.1. Study Design and Participants

This multicentre, longitudinal, prospective study analysed a cohort of patients admitted to two centres of the Don Carlo Gnocchi Foundation between September 2020 and April 2023 (NUTRISTROKE study, clinical study identifier: NCT04923165). Patients were evaluated at admission (T0) and after a 6-week rehabilitation program (T1).

We recorded demographic, anamnestic, and clinical data at admission (T0). Disease burden was measured with the 56-point Cumulative Illness Rating Scale (CIRS) [39].

The following criteria were used to include the patients: (i) first ischemic or haemorrhagic stroke, confirmed by magnetic resonance imaging (MRI) or computed tomography (CT); (ii) age between 18 and 85; (iii) less than six months since the stroke insult; (iv) adequate cognitive and language abilities to comprehend the instructions for administering the assessment scales and to sign the informed consent.

The exclusion criteria were the following: (i) a previous stroke; (ii) behavioural and cognitive disorders and/or reduced compliance interfering with active therapy or with understanding and signing informed consent.

Moreover, at enrolment and during evaluation, patients were not infected with SARS-CoV-2.

The study design was approved by the Ethical Committee of the Don Carlo Gnocchi Foundation, Milan, Italy, on October 14, 2020 (FDG_6_14/10/20). All patients gave their written informed permission after being fully informed about the study’s objectives and treatment procedures.

### 2.2. Biochemical Analyses 

The blood samples of patients were collected in the early morning (7:30–9:00 a.m.) after an overnight fast to standardise the assessment of those biochemical variables that are affected by the circadian cycle and food intake. Serum samples were separated by centrifugation (3000 rpm, 10 min, and 4 °C), divided into 0.5 mL aliquots, and rapidly stored at −80 °C. Subjects’ and reference samples were thawed just before the assays. All the analyses were performed in duplicate both at T0 and T1 and tested on an integrated analytical photometer (Diacron International srl, Grosseto, Italy).

#### 2.2.1. Oxidative Stress Analysis

The colorimetric determination of hydroperoxides was assessed by the d-ROMs test (Diacron International srl, Grosseto, Italy). In this test, hydroperoxides contained in the biological sample, in the presence of iron, generate alkoxyl and peroxyl radicals, according to Fenton’s reaction. The radicals, reacting with a chromogenic mixture, oxidise it and transform it into a photometrically measurable coloured derivative. The values are expressed in arbitrary units (UCARR), with 1 UCARR corresponding to 0.08 mg/100 mL of hydrogen peroxide [40]. The normal values are between 250 and 300 UCARR. Values higher than 300 UCARR indicate progressively increasing levels of oxidative stress: borderline range (301–320 UCARR), low level of oxidative stress (321–340 UCARR), middle level of oxidative stress (341–400 UCARR), high level of oxidative stress (401–500 UCARR), and very high level of oxidative stress (>500 UCARR). The reproducibility of the test reported as CV% is 1.72.

The biological antioxidant power (BAP) test measures the amount of endogenous (e.g., bilirubin, uric acid, and proteins) and exogenous (e.g., ascorbate, tocopherols, carotenoids, and flavonoids) substances in plasma that have the potential to be antioxidants and can counteract radical species (Diacron International srl, Grosseto, Italy). The biological antioxidant power of the plasma barrier corresponds to the ability of all antioxidants to reduce a coloured solution of ferric ions (Fe^3+^) to ferrous ions (Fe^2+^), resulting in a photometrically detectable decolorization. The reference ranges for BAP are as follows: optimal status (>2200 µmol/L), borderline status (2200–2000 µmol/L), slight deficiency status (1999–1800 µmol/L), deficiency status (1799–1600 µmol/L), and high deficiency status (<1600 µmol/L). The reproducibility of this test, expressed as CV%, is 2.32.

Moreover, we analysed the thiol groups, which are a significant component of the antioxidant plasma barrier, using the -SHp test (Diacron International srl, Grosseto, Italy) [41]. The -SHp test is based on the ability of thiol groups present in the biological sample to develop a photometrically detectable coloured complex when they react with 5,5-dithiobis-2-nitrobenzoic acid, dissolved in a chromogenic blend. In particular, the -SHp test was used to quantify the concentration of total thiols (e.g., albumin, lipoic acid, and glutathione) present in plasma. The normal range of the -SHp is >450 µmol/L. The reproducibility of this test, expressed as CV%, is 3.47.

We calculated two indices to assess the relative antioxidant capacity, namely the antioxidant capacity normalised for total circulating hydroperoxides: (i) the OSI index, as published in our previous work [24]; (ii) the thiol antioxidant capacity SH-OSI index (calculated as the ratio -SHp/d-ROMs). To our knowledge, this ratio has never before been described in the literature. The cut-off of normality was set at 7.3 for the OSI index [24]. For the SH-OSI index, we calculated the range of normality starting from the reference cut-off reported for the -SHp test and the d-ROMs test from Diacron International srl (Diacron International srl, Grosseto, Italy). This assumption yields a range of 1.3–2.6, with values below the range indicating an inadequate relative thiol antioxidant capacity.

#### 2.2.2. Hematochemical Analysis

Albumin levels were measured by a bromocresol colorimetric assay (Diacron International srl, Grosseto, Italy) [42]; glucose was assessed by an oxidase/peroxidase system (Diacron International srl, Grosseto, Italy). Total cholesterol was measured by means of oxidation from a cholesteroxidase to cholest-4-en-3-one [43], while direct HDL cholesterol was assessed by a transformation of the HDL portion into a quinone derivative [44] (Diacron International srl, Grosseto, Italy); triglycerides were measured by a peroxidase-coupled method [45] (Diacron International srl, Grosseto, Italy).

### 2.3. Rehabilitation Treatment 

Patients completed a rehabilitation treatment that included traditional physical therapy, which was carried out for 45 min each day, 6 days a week. The rehabilitation treatment consisted of passive, active-assisted, and active mobilisations, exercises for muscle strength recovery, stretching, functional and task-oriented training, proprioceptive exercises, postural passages and transfers, sitting and standing training, motor coordination and balance training, walking training, and activities of daily living recovery training. Additionally, each patient underwent robotic treatment of the upper limb five times each week for 45 min at a time. The following robotic devices were employed: Motore (Humanware Srl, Pisa, Italy), Amadeo, Diego, and Pablo (Tyromotion GmbH, Graz, Austria), as detailed in previous studies [46,47]. Upper-limb robotic therapy allowed both motor and cognitive tasks, thanks to the visual and audible input associated with the equipment.

### 2.4. Activity of Daily Living, Endurance and Strength Assessment 

A functional independence evaluation was conducted both at admission and after 6 weeks of rehabilitation treatment (T1) to assess the effect of the rehabilitation treatment. To measure the performance in Activity of Daily Living (ADL), the modified Barthel Index (mBI) was employed, which is an ordinal scale ranging from 0 to 100, with a lower score associated with higher disability [48]. The mBI contains 10 items measuring the patient’s ability to perform different activities (feeding, personal hygiene, dressing, bathing, bladder control, bowel control, toilet transfers, stair climbing, and ambulation/wheelchair).

The walking endurance was assessed by a 6-min walk test (6MWT) in patients who could walk. It assesses distance walked over 6 min as a sub-maximal test of aerobic capacity and endurance [49].

A quantitative measure of isometric muscular strength of the hand and forearm affected and not affected by hemiparesis was assessed with the Hand Grip test using force production expressed in kilogrammes (Citec CIT Tecnics, Haren, The Netherlands). The mean of three trials was registered as the measure of hand strength.

### 2.5. Statistical Analysis

Demographic and clinical characteristics of patients were presented using numerical data, expressed as the mean and standard deviation, and categorical data, expressed as counts and percentages.

Analysis of normality was performed with the Shapiro–Wilk test, and non-parametric tests were employed accordingly.

Biochemical, demographic, and clinical data were analysed disaggregated by gender and for stroke type using the Mann–Whitney U test or the chi-squared test.

To investigate the changes induced by the rehabilitation treatment on oxidative stress parameters and functional parameters (mBI, Hand Grip test data, 6MWT), values at T0 and T1 were compared using the Wilcoxon signed-rank test. The McNemar test was employed to analyse the frequency distribution of paired data.

Finally, Spearman’s rank correlation coefficients were used to examine the relationship between oxidative stress parameters, demographic and clinical parameters, and rehabilitation outcomes at both timepoints. Spearman’s partial correlation analyses were performed to include the effect of variables (age and days from stroke event) on the significant correlations found.

For all the statistical analyses, a *p*-value below 0.05 was considered significant. Statistical analysis was performed using SPSS (IBM SPSS Statistics for Windows, Version 28.0. Armonk, NY, USA: IBM Corp).

## 3. Results

### 3.1. Participants and Baseline Characteristics

We screened 140 patients, and 119 met the inclusion criteria and were enrolled in the study; 10 patients did not complete the follow-up due to clinical adverse conditions. Finally, 109 patients (51 women, 58 men, mean age 69 ± 11 years) were evaluated at baseline (T0) and after a six-week rehabilitation treatment (T1).

Baseline characteristics (demographic and clinical features and disability assessment) are reported for the whole group, for women and for men, in Table 1. The sample was homogeneous between genders except for age, weight, height, HDL cholesterol, and the Hand Grip test evaluated both in the hemiparetic arm (affected) and in the healthy arm (unaffected). We did not observe any differences between ischemic and haemorrhagic subjects for the demographic and clinical characteristics of the subjects and for the functionality, strength, and endurance assessments.

### 3.2. Oxidative Stress Parameters at Baseline

Oxidative stress parameters (d-ROMs, BAP, -SHp; OSI index; and SH-OSI index) at baseline (T0) are reported for the whole group and stratified by gender (Table 2). We did not find differences in oxidative stress parameters between women and men. We observed no significant differences in the parameters of systemic oxidative status between ischemic and haemorrhagic patients.

**Figure 1:**
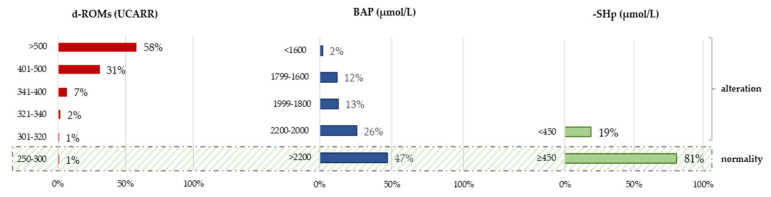
The histograms show at baseline (T0) the percentage of patients for each reference range of d-ROMs, BAP, and the -SHp. The reference ranges for the d-ROMs are as follows: normal (250–300 UCARR), borderline (301–320 UCARR), low level (321–340 UCARR), middle level (341–400 UCARR), high level (401–500 UCARR), and very high level (>500 UCARR). The reference ranges for the BAP are as follows: optimal status (>2200 µmol/L), borderline status (2200–2000 µmol/L), slight deficiency status (1999–1800 µmol/L), deficiency status (1799–1600 µmol/L), and high deficiency status (<1600 µmol/L). The reference range for the -SHp are as follows: normal levels (>450 µmol/L), insufficient levels (<450 µmol/L). The dashed area refers to the range of normality.

At admission, a significant percentage of patients had high (31%) and very high (58%) levels of hydroperoxides (d-ROMs). In terms of the antioxidant component, 53% of the recruited patients did not have optimal levels of total antioxidants (BAP), while 19% of patients had inadequate levels of the thiol component (-SHp) (Figure 1). In addition, 90% of the patients had an OSI index value lower than the normality cut-off (7.3), and 66% had an SH-OSI index below the estimated normal range of 1.3–2.6.

At T0, -SHp was negatively correlated with age (r = −0.359, *p* <0.001) and positively correlated with the distance from the acute event (r = 0.462, *p* <0.001); the same significant correlations were observed for the SH-OSI index (r = −0.201, *p* = 0.007; 0.371, *p* = 0.003).

Table 3 shows Spearman’s partial correlations, conditioned on age and distance from the acute event, between oxidative stress parameters and ADL (mBI) and strength assessments at T0. Hydroperoxide levels negatively correlated with the mBI, while the antioxidant thiol component showed a positive correlation with the mBI and the Hand Grip test values of the unaffected and affected arms. Regarding the oxidative stress indices, both the OSI index and the SH-OSI index were positively correlated with the mBI and the Hand Grip test of the unaffected arm, while SH-OSI also correlated with the Hand Grip test of the affected arm.

Finally, no meaningful correlations were found between the oxidative stress parameters and the biochemical parameters analysed in this study.

### 3.3. Rehabilitation Effect 

As expected, the clinical outcome measures significantly improved after the rehabilitation treatment, in particular, the mean scores of the mBI (42 ± 22 vs. 59 ± 27, *p* < 0.001) and the 6MWT (296 ± 122 vs. 241 ± 153, *p* = 0.011). It is important to highlight that at baseline, only 17 subjects performed the 6MWT, but after rehabilitation, the number of subjects able to perform the test was 38. The Hand Grip test values assessed on the arm affected by hemiparesis were also higher at T1 than at T0 (6.2 ± 8.1 vs. 7.1 ± 8.5, *p* = 0.014), but not in the healthy arm (19.8 ± 11.6 vs. 19.8 ± 11.2, *p* = 0.568).

**Figure 2:**
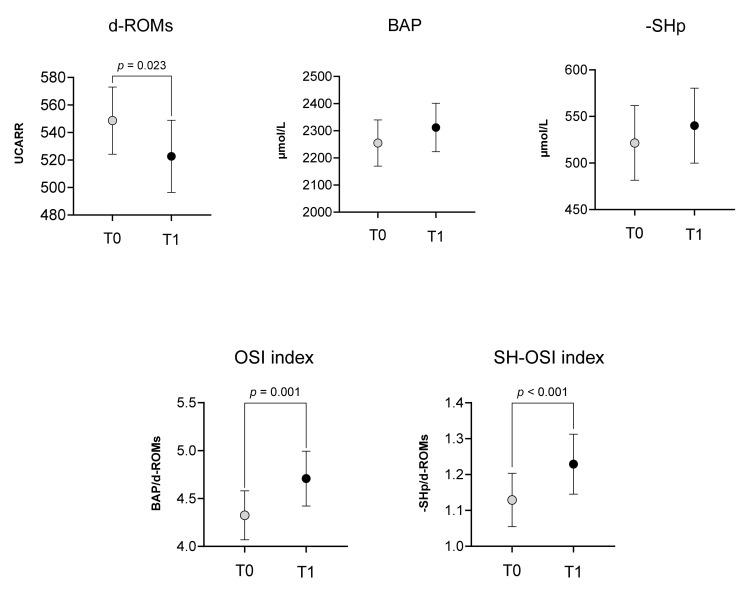
Changes in oxidative stress parameters between baseline (T0) and at the end of the rehabilitation program (T1). Mean bars and 95% CI are reported. The *p*-value refers to the statistically significant differences found by the Wilcoxon signed-rank test.

Figure 2 reports the comparison of oxidative stress parameters between T0 and T1. Noteworthy, after the rehabilitation treatment, we observed a significant decrease in d-ROMs (549 ± 126 vs. 523 ± 148, *p* = 0.023), as well as a significant improvement in the OSI index (4.3 ± 1.3 vs. 4.7 ± 1.5, *p* = 0.001) and SH-OSI index (1.1 ± 0.4 vs. 1.2 ± 0.4, *p* < 0.001). On the contrary, we did not find any difference in BAP (2255 ± 440 vs. 2312 ± 460, *p* = 0.289) and -SHp values (587 ± 146 vs. 603 ± 149, *p* = 0.204).

However, the McNemar test for related samples found that 45% of patients with inadequate thiol components at T0 reached normal values at T1 (*p* < 0.021).

The difference in values between T1 and T0 was higher than the CV% values stated in the tests.

In addition, a negative partial correlation between the 6MWT and d-ROMs (r = −0.422, *p* = 0.012) and a positive partial correlation between the 6MWT and SH-OSI were seen at T1 in 38 walking subjects (r = 0.431, *p* = 0.009).

## 4. Discussion

This study showed a significant alteration of systemic oxidative stress status in subjects with subacute stroke, both on admission and after six weeks of rehabilitation treatment.

The hydroperoxides, an ROS component, measured by the d-ROMs test at T0 were high and very high in 31% and 58% of patients, respectively (Figure 1). The systemic oxidative status has been investigated in previous studies, showing a very high imbalance with respect to healthy controls, not only immediately after the stroke event [20,25,50] but also in the subacute [24,25,51] and chronic phases of stroke [27,28].

The high levels of systemic oxidative stress observed in post-stroke patients may be indirect indicators of an altered condition in the brain. Following an ischaemic event or haemorrhagic brain injury, biochemical and molecular changes occurring at the neuronal level contribute to the activation of a series of pathological pathways, including the massive production of reactive oxygen species (ROS), which contribute to worsening the neural impairment [11]. Brain tissue is particularly vulnerable to damage caused by ROS due to high concentrations of peroxidizable lipids, low levels of antioxidant enzymes, high oxygen consumption, high levels of iron that can act as pro-oxidants, entering in Fenton’s reactions, and the presence of neurotransmitters that can increase intracellular calcium [12,14].

Moreover, even if the mechanism of ROS action in the brain is not yet fully understood, the role of ROS as mediators in the impairment of the blood–brain barrier (BBB) is now recognised [52,53]. Under physiological conditions, the BBB is a physical and metabolic barrier that promotes cerebral homeostasis. After the stroke event, the increase in ROS in the brain can modify the BBB, even at the level of the tight junctions (TJs); this involves an increase in permeability and an alteration in the molecules’ influx from the systemic circulation into the brain’s extracellular space, with serious clinical consequences such as brain oedema and haemorrhagic transformation. In this context, it is possible that the presence of systemic oxidative stress could contribute to further BBB and neuronal damage [54,55].

The ROS, both at systemic and brain levels, contribute to neuroinflammatory processes, mitochondrial dysfunction, and cell death mechanisms, leading to subsequent brain damage that may affect recovery [16,49,50]. Furthermore, the increase in ROS has been linked to a decrease in synaptic long-term potentiation, synaptic signalling, and brain plasticity mechanisms [16], which can affect functional recovery [51,52,53].

The BAP test showed that 53% of our sample had changed biological antioxidant power at T0. This meant that most of the people in the sample did not have the best antioxidant capacity, which is different from our previous study [24]. On the other hand, the antioxidant thiol component, tested with the -SHp test, was below the reference range in only 19% of subjects at T0.

The relative antioxidant capacity, assessed through the OSI index, was below the cut-off of normality in 90% of subjects, while the SH-OSI index was below the estimated range of normality in 66% of subjects, pointing out that both total antioxidants and thiol components are inadequate in counteracting the circulating hydroperoxides.

After the six weeks of rehabilitation, the oxidative stress panel showed a change, although it was not sufficient to overcome the redox imbalance. In fact, at T1, the mean values of d-ROMs significantly decreased with respect to T0, while the OSI and SH-OSI indices increased (Figure 2), even if they remained outside of normality ranges. There were no significant changes in antioxidant status variables, even if we observed an improvement trend. Nonetheless, 45% of patients with suboptimal thiol antioxidant levels at T0 achieved normal values at T1.

It is important to note that the elevated mean age of our sample (69 ± 11 years) might compromise the antioxidant defences. Indeed, during the ageing process, parallel to an increase in ROS, there may be a decrease both in exogenous antioxidants assumed by diet and in the production of endogenous factors [56,57]. In this regard, at both T0 and T1, we found a negative correlation between the -SHp and the SH-OSI index with age, indicating that thiol groups are more sensitive to physiological changes.

Furthermore, the -SHp and SH-OSI correlated positively with distance from the acute event, implying that patients evaluated more distant from the stroke insult had a higher level of the thiol antioxidant component. It is possible to suppose that an improvement in physiological and/or clinical conditions is related to an improvement in thiol compounds. In fact, Musumeci et al. showed that in acute stroke patients, thiol concentrations were significantly lower than in healthy controls and significantly lower in the subgroup of patients with the worst clinical outcome [34]. The authors hypothesised that the body employed reduced sulfhydryl groups (thiol) to neutralise the abnormal production of free radicals; as a result, lower levels could be detected in patients’ serum immediately after stroke. In our sample, we investigated subacute post-stroke patients, and the reduction in the thiol component may be related to both advanced age and distance from the insult.

After the six-week rehabilitation program, patients ameliorated their functional status: as expected, performance in ADL, strength assessed on the arm with hemiparesis, and walking endurance increased. The correlations between oxidative stress parameters and rehabilitation outcomes, corrected for age and distance from the acute event, were interesting. The d-ROMs showed a negative correlation with mBI at T0; moreover, hydroperoxide levels had a negative correlation with Hand Grip measurements of the unaffected arm and the 6MWT at T1. Considering the antioxidant panel, the -SHp correlated positively with the mBI and the Hand Grip test of healthy arms at T0. The oxidative stress indices showed similar positive correlations (Table 3). From these results, we can suppose that variations in oxidative stress parameters can have some relationship with a better functional outcome, as seen previously [28]. Exercise can, in fact, contribute to the improvement of antioxidant defences and the reduction in oxidative stress [51,58,59]. A study conducted on elderly individuals showed that the activity levels of antioxidant enzymes were higher in subjects who practiced physical activity regularly, compared to sedentary subjects of the same age [55]. On the contrary, growing evidence shows that ROS may contribute to muscle fatigue [60,61,62].

The positive correlation observed in our study between the -SHp and the Hand Grip test assessed on the healthy arm indicates that higher levels of thiol groups can be related to the mechanism of muscle strength. It has been shown that the intake of N-acetylcysteine, which promotes the synthesis of the antioxidant glutathione, reduces cellular oxidation and delays muscle fatigue [63,64].

Further research should be conducted in the future to examine the potential efficacy of antioxidant supplementation or a particular diet in mitigating the detrimental effects of stroke-related oxidative stress, thereby promoting recovery and preventing further neural damage. To our knowledge, in fact, there are still few studies that have investigated in humans the effects of therapies for reducing oxidative stress in the subacute phase of stroke. A very recent study found that giving rodents glucagon-like peptide-1 receptor agonists (GLP-1RAs) lowers the amount of d-ROMs in their blood after they have had a stroke. The administration of these drugs during the subacute phase has been shown to improve functional recovery in post-stroke mice [65]. Similar results were observed in patients with type 2 diabetes, giving some interesting future areas of study [66,67].

An advantage of this study is that we employed an integrated analytical panel test that does not require high-skill experience or specialised laboratories, allowing easy monitoring of hospitalised patients. Furthermore, the reported data adheres to “gold standard” methodologies, which enable very high levels of accuracy [68,69]. Another strength is that this study was performed in two different centres.

On the other hand, a limitation of this study was that we did not measure biomarkers of inflammation, which would merit future investigation due to their close interdependence with oxidative stress.

## 5. Conclusions

These data suggest the need to monitor the systemic oxidative state in post-stroke patients, not only because it appears to be severely altered and thus potentially dangerous for neuronal cells, but also because of its possible interrelation with functional recovery. In this regard, periodic analysis of pro-oxidant and antioxidant molecules in these patients is especially suggested. Further studies will be necessary to investigate in more detail the oxidative stress parameters that are more involved in lipid peroxidation, which is strongly associated with neuronal damage, as well as the possible contribution of antioxidant supplements or a particular diet to overcoming the oxidative imbalance reported in this study.

## Figures and Tables

**Table 1:** Baseline (T0) characteristics of the whole group (n = 109), women (n = 51) and men (n = 58). Data are reported as mean ± standard deviation or number (%). *p*-values refer to the Mann–Whitney U test or the chi-squared test, as appropriate.

Baseline Characteristics	Whole Group (n = 109)	Women (n = 51)	Men (n = 58)	*p*-Value
Age (years)	69 ± 11	72 ± 9	67 ± 12	0.027 *
Anthropometric value				
Weight (kg)	69 ± 17	62 ± 13	75 ± 18	<0.001 ***
Height (m)	1.7 ± 10.2	1.6 ± 6.7	1.7 ± 10.2	<0.001 ***
BMI (kg/m^2^)	24.9 ± 4.8	24.2 ± 5.1	25.5 ± 4.4	0.055
Index stroke type				
Ischemic	86 (79%)	42 (82%)	44 (76%)	0.412
Haemorrhagic	23 (21%)	9 (18%)	14 (24%)	
Affected side				
Right	58 (53%)	27 (53%)	31 (56%)	0.961
Left	51 (47%)	24 (47%)	27 (47%)	
Smoking	50 (46%)	19 (37%)	31 (53%)	0.103
Comorbidities				
Hypertension	93 (85%)	44 (86%)	49 (84%)	0.797
Type 2 diabetes	28 (26%)	12 (24%)	16 (28%)	0.633
Dyslipidaemia	45 (41%)	22 (43%)	23 (40%)	0.716
Heart disease	16 (17%)	4 (9%)	12 (23%)	0.082
Dysphagia	29 (27%)	16 (31%)	13 (22%)	0.451
Cumulative Illness Rating Scale (CIRS)				
CIRS severity	2.2 ± 0.4	2.2 ± 0.4	2.1 ± 0.4	0.066
CIRS comorbidity	5.1 ± 2.0	5.3 ± 1.9	4.9 ± 2.2	0.285
Days from stroke onset to enrolment	87 ± 57	97 ± 62	77 ± 52	0.079
Activity of Daily Living (ADL) assessment				
Modified Barthel Index (mBI)	42 ± 22	37 ± 19	46 ± 24	0.094
Endurance motor assessment	(n = 17)	(n = 6)	(n = 11)	
6 min walk test (6MWT, m/min)	296 ± 122	249 ± 150	321 ± 102	0.313
Strength of arms				
Hand Grip test of unaffected arm (kg)	19.8 ± 11.6	15.1 ± 8.7	23.9 ± 12.4	<0.001 ***
Hand Grip test of affected arm (kg)	6.2 ± 8.1	3.9 ± 8.2	8.2 ± 9.6	0.015 *
Ematochemical analyses				
Glucose (mg/dL)	111 ± 39	116 ± 45	105 ± 33	0.294
Cholesterol (mg/dL)	126 ± 37	133 ± 41	119 ± 31	0.207
HDL cholesterol (mg/dL)	53 ± 17	57 ± 15	49 ± 18	0.005 **
Triglycerides (mg/dL)	123 ± 58	120 ± 61	126 ± 56	0.372

* *p*-value < 0.05; ** *p*-value < 0.01; *** *p*-value < 0.001.

**Table 2:** Oxidative stress analyses at baseline (T0). Data are reported as mean± standard deviation. *p*-values refer to the Mann–Whitney U test.

Oxidative Stress Parameters	Whole Group (n = 109)	Women (n = 51)	Men (n = 58)	*p*-Value
d-ROMs (UCARR)	549 ± 126	562 ± 139	537 ± 113	0.498
BAP (µmol/L)	2255 ± 440	2267 ± 491	2244 ± 394	0.810
-SHp (µmol/L)	587 ± 146	580 ± 131	593 ± 158	0.987
OSI index (BAP/d-ROMs)	4.3 ± 1.3	4.3 ± 1.4	4.4 ± 1.3	0.585
SH-OSI index (-SHp/d-ROMs)	1.1 ± 0.4	1.1 ± 0.4	1.2 ± 0.4	0.598

**Table 3:** Correlations between oxidative stress parameters and the ADL performance (modified Barthel Index, mBI) and the Hand Grip test of the unaffected and affected arm. *p*-values refer to the Spearman’s partial correlations conditioned on age and distance from the acute event.

	mBI		Hand Grip Test Unaffected Arm (kg)		Hand Grip Test Affected Arm (kg)	
Oxidative Stress	Spearman’s Rho	*p*	Spearman’s Rho	*p*	Spearman’s Rho	*p*
d-ROMs (UCARR)	−0.300 **	0.003	−0.091	0.367	−0.138	0.170
BAP (µmol/L)	0.013	0.898	−7.839 × 10^−4^	0.994	−0.045	0.657
-SHp (µmol/L)	0.244 *	0.103	0.326 ***	<0.001	0.201 *	0.044
OSI index (BAP/d-ROMs)	0.265 **	0.007	0.053	0.596	0.113	0.262
SH-OSI index (SHp/d-ROMs)	0.324 ***	<0.001	0.276 **	0.005	0.258 **	0.009

mBI, modified Barthel Index; * *p*-value < 0.05; ** *p*-value < 0.01; *** *p*-value < 0.001.

## Data Availability

The data supporting the findings of this study are available from the corresponding author upon reasonable request.
